# A56 THE ROLE OF THE STERILE ALPHA MOTIF (SAM) POINTED DOMAIN ETS FAMILY TRANSCRIPTION FACTOR (SPDEF) ON GOBLET CELL BIOLOGY AND INTESTINAL MUCUS PRODUCTION IN THE CONTEXT OF PARASITIC HOST DEFENSE.

**DOI:** 10.1093/jcag/gwae059.056

**Published:** 2025-02-10

**Authors:** T Seto, Z Haider, J Grondin, H Wang, S Haq, S Banskota, W I Khan

**Affiliations:** Pathology and Molecular Medicine, McMaster University, Hamilton, ON, Canada; Pathology and Molecular Medicine, McMaster University, Hamilton, ON, Canada; Pathology and Molecular Medicine, McMaster University, Hamilton, ON, Canada; Pathology and Molecular Medicine, McMaster University, Hamilton, ON, Canada; Pathology and Molecular Medicine, McMaster University, Hamilton, ON, Canada; Pathology and Molecular Medicine, McMaster University, Hamilton, ON, Canada; Pathology and Molecular Medicine, McMaster University, Hamilton, ON, Canada

## Abstract

**Background:**

The intestinal mucus layer in the gastrointestinal (GI) tract acts as a physical protective barrier against luminal contents and potential invaders. Goblet cells, a specialized subset of intestinal epithelial cells, play a large role in synthesizing and secreting mucins, which are the structural building blocks of the intestinal mucus layer. The terminal differentiation and maturation of these goblet cells are regulated by sterile alpha motif (SAM) pointed domain ETS family transcription factor (SPDEF). Goblet cell biology and the intestinal mucus barrier is one of the most effective host response mechanisms against enteric parasitic infections, including *Trichuris muris*. However, no study has evaluated the relationship between SPDEF, goblet cell biology, and mucus production in the context of parasitic host defense.

**Aims:**

To identify the relationship between SPDEF, goblet cell biology, and mucus production during *T. muris* infection and how this contributes to parasitic host defence.

**Methods:**

For infection experiments, BALB/c mice were gavaged with ~300 *T. muris* eggs in 200 µl autoclaved distilled water. To explore the role of SPDEF in goblet cell and mucin responses during *T. muris* infection, SPDEF^*-/-*^ and SPDEF^*+/+*^ mice were administered ~300 *T. muris* eggs and were sacrificed at days 14 and 21 post-infection (p.i) along with uninfected controls. Periodic Acid Schiff (PAS) staining was used to quantify goblet cells and immunofluorescence was used to identify Muc2^+^ and SPDEF^+^ cells. Enzyme-linked immunosorbent assays were used to evaluate Th2 cytokines, and qPCR was used to evaluate *Muc2* and *SPDEF* expression.

**Results:**

BALB/c mice infected with *T. muris* displayed increased *SPDEF* expression. On days 14 and 21 p.i., SPDEF^*-/-*^ mice harboured significantly more worms than their SPDEF^*+/+*^ counterparts. These mice also displayed diminished levels of IL-4 and IL-13 at day 14 p.i. as well as decreased PAS^+^ and MUC2^+^ goblet cell numbers. Compared to SPDEF^*+/+*^ mice, on day 21 p.i., IL-4 was increased in infected SPDEF^*-/-*^ mice, whereas levels of PAS^+^ and MUC2^+^ goblet cells retained a similar pattern to that of day 14 p.i. Interestingly, the number of PAS^+^ goblet cells, MUC2^+^ cells and *Muc2* expression significantly increased after infection in *SPDEF*^*+/+*^ mice at day 14 and day 21 p.i.

**Conclusions:**

Together, these results suggest that SPDEF is an important regulator of goblet cell function and mucus production during *T. muris* infection, ultimately contributing to parasitic host defense.

**Funding Agencies:**

